# Patient-Reported Outcomes and Quality of Life After Laparoscopic Pectopexy

**DOI:** 10.3390/jcm14176318

**Published:** 2025-09-07

**Authors:** Anna Pitsillidi, Georgios Grigoriadis, Laura Vona, Guenter Noé, Angelos Daniilidis

**Affiliations:** 1 Department of OB/GYN, Rheinland Klinikum Neuss, Preußenstrasse 84, 41464 Neuss, Germany; guenter.noe@uni-wh.de; 2 1st University Department in Obstetrics and Gynecology, Papageorgiou General Hospital, School of Medicine, Aristotle University of Thessaloniki, 546 43 Thessaloniki, Greece; drgeorgiosgrigoriadis@gmail.com (G.G.); angedan@hotmail.com (A.D.); 3 Department of Medical and Surgical Sciences, Institute of Obstetrics and Gynaecology, University of Foggia, 71122 Foggia, Italy; laura.vona@unifg.it; 4 Department of OB/GYN, University of Witten Herdecke, 58448 Witten, Germany

**Keywords:** laparoscopy, pectopexy, pelvic organ prolapse, quality of life, sexual life

## Abstract

**Background:** Pelvic organ prolapse (POP) significantly impairs patients’ quality of life, especially in urinary, bowel, and sexual domains. While laparoscopic sacrocolpopexy (LS) is the current gold standard for apical prolapse repair, it is associated with certain complications. Laparoscopic pectopexy (LP), a newer technique utilizing the iliopectineal ligament for apical suspension, may offer improved outcomes with fewer adverse effects. This scoping review aimed to evaluate patient-reported outcomes (PROs) and quality of life (QoL) following LP and compare its effectiveness to other established surgical approaches. **Methods:** A scoping review was conducted in accordance with PRISMA-ScR guidelines. Searches of PubMed, Scopus, and Web of Science databases were performed through June 2025. Eligible studies included randomized controlled trials, prospective and retrospective cohorts, and case series that reported PROs following LP. Data on validated QoL tools (e.g., P-QOL, PFDI-20, PFIQ-7, FSFI, PISQ-12), surgical technique, and follow-up duration were extracted. Due to heterogeneity in the study design and outcomes, findings were synthesized qualitatively. **Results:** Thirteen studies including a total of 742 patients met the inclusion criteria. Across all included studies, LP was associated with significant improvements in QoL metrics, including urinary and sexual function, and overall patient satisfaction. Tools such as PFDI-20, FSFI, PISQ-12, and PGI-I consistently showed postoperative improvement (*p* < 0.05). Comparative studies demonstrated that the outcomes for LP were similar or superior to those of sacrocolpopexy, sacrospinous fixation, or sacrohysteropexy, particularly regarding sexual function. **Conclusions:** LP is an effective surgical alternative for apical POP repair, offering significant improvements in patient-reported quality of life and functional outcomes. Its favorable safety profile and comparable efficacy to traditional methods make it a compelling option, particularly for patients with contraindications to sacral dissection. Findings are limited by small and heterogeneous studies, short follow-up, and potential publication and language biases. Further prospective studies with long-term follow-up periods are necessary to confirm these findings and refine patient selection criteria.

## 1. Introduction

Pelvic organ prolapse (POP) is a prevalent condition that affects up to 50% of parous women and significantly impairs their quality of life, particularly in areas related to urinary, bowel, and sexual function [1]. Effective treatment is crucial to prevent social isolation, particularly among middle-aged and older women. Sacrocolpopexy is widely recognized as the gold standard surgical approach for apical prolapse, primarily due to its ability to restore pelvic support while maintaining the natural vaginal axis [2]. Minimally invasive variants, such as laparoscopic and robotic-assisted sacrocolpopexy, have further improved patient outcomes by reducing surgical morbidity, minimizing postoperative pain, and shortening recovery time. However, these techniques are not without drawbacks. Postoperative complications such as constipation, pelvic pain, and mesh-related discomfort have been reported [3]. In an effort to overcome these limitations, a novel technique known as laparoscopic pectopexy (LP) was introduced by Noé et al. [4]. This approach utilizes the iliopectineal (Cooper’s) ligament for apical suspension, thereby avoiding dissection near the sacral promontory and reducing the risk of associated complications [4,5].

As surgical outcomes increasingly prioritize patient-centered measures, it is essential to evaluate not only anatomical success but also improvements in functional status and overall well-being. Patient-reported outcome measures (PROMs), including validated quality of life (QoL) questionnaires, provide valuable insights into the real-world impact of prolapse surgery on urinary, bowel, and sexual function. Recent systematic reviews and meta-analyses have emphasized the growing importance of these outcomes in assessing surgical efficacy for apical prolapse. Notably, a meta-analysis comparing laparoscopic pectopexy with laparoscopic sacrocolpopexy found that both techniques offer comparable improvements in functional outcomes, with pectopexy associated with shorter operative times and reduced postoperative hospitalization [6]. This scoping review aims to synthesize the current literature on QoL following LP, examining how this technique affects functional outcomes and patient satisfaction, and drawing comparisons with other established surgical approaches where available. In addition to standardized QoL instruments, some studies have employed alternative methods, such as goal-oriented measures, which may provide complementary insights into individualized, patient-centered outcomes.

## 2. Materials and Methods

### 2.1. Search Strategy

**Figure 1:**
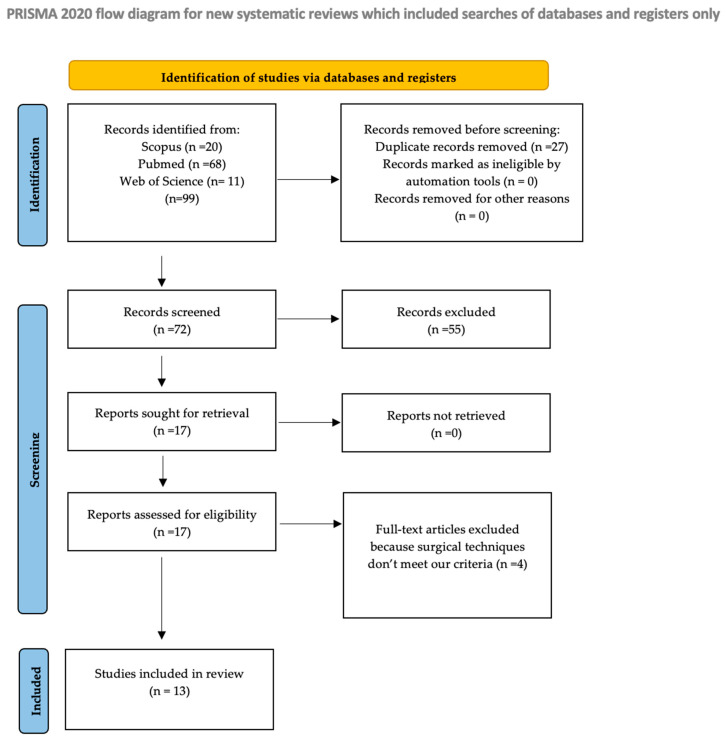
Flow diagram.

This scoping review was conducted in accordance with the PRISMA-ScR (Preferred Reporting Items for Systematic Reviews and Meta-Analyses extension for Scoping Reviews) guidelines (see Supplemental Table S1: PRISMA-ScR) [7]. A systematic literature search was independently carried out by two reviewers (A.P. and G.G.) across the Web of Science, Scopus, and MEDLINE (via PubMed) databases. A combination of free-text terms and MeSH headings was used to identify relevant studies. Search terms such as ‘laparoscopic pectopexy,’ ‘pectopexy,’ ‘pelvic organ prolapse,’ ‘quality of life,’ and ‘patient-reported outcomes’ were combined using Boolean operators (OR within each concept and AND between different concepts). The database search yielded records up to June 2025, with no limitations regarding publication date. While studies published in 2024–2025 were retrieved and screened, none met the eligibility criteria. These records were excluded primarily due to an inappropriate study design, absence of relevant outcomes, or lack of alignment with the review question. Consequently, the most recent included studies up to 2023. The study selection process is illustrated in the PRISMA flow chart (Figure 1).

### 2.2. Eligibility Criteria

Study designs eligible for inclusion comprised retrospective analyses, observational case series, prospective cohort investigations, randomized clinical trials (RCTs), and controlled prospective studies. Only peer-reviewed, full-text publications written in English were evaluated. In contrast, sources such as systematic reviews, meta-analyses, editorials, and conference abstracts were excluded. Nonetheless, the reference sections of relevant reviews were screened to identify additional eligible studies. The exclusion criteria also included studies with unclear methodology, incomplete or poor-quality data, non-quantifiable outcomes, or a lack of patient-reported outcomes. Articles whose full text was not published in English, as well as case reports, studies without Patient-Reported Outcomes data, animal studies, and non-laparoscopic techniques or techniques without mesh, were excluded.

### 2.3. Data Acquisition and Risk of Bias

All articles retrieved from the database search were initially assessed based on full-text availability, abstract content, authorship, article title, reference details, and year of publication. Two independent reviewers (A.P. and G.G.) manually identified and eliminated duplicate records. In the subsequent screening phase, both reviewers independently examined abstracts and titles to exclude studies that were clearly irrelevant to the research objective. Full-text evaluation was then performed independently for all potentially eligible publications. Discrepancies between the two reviewers were resolved through discussion and consensus; a third reviewer (L.V.) was available for arbitration, although this was not ultimately required. A formal standardized screening tool was not employed, but predefined eligibility criteria were applied consistently. Inter-rater agreement statistics (e.g., Cohen’s kappa) were not calculated; however, reliability was ensured through independent assessment and the consensus-based resolution of disagreements. The methodological rigor of the selected studies was evaluated using the Joanna Briggs Institute (JBI) Critical Appraisal Checklists for Case Reports and Clinical Trials (Supplemental Table S2).

### 2.4. Data Synthesis and Statistical Analysis

After completing the screening process, 13 manuscripts were deemed eligible and included in the review (Table 1). A scoping review approach was adopted instead of a systematic review and meta-analysis because the available studies presented substantial heterogeneity in their study designs, outcome measures, and reporting formats. The included evidence encompassed diverse study types (e.g., cross-sectional, cohort, RCT), each addressing different aspects of the research question. Given this variability, a systematic synthesis of effect estimates was not feasible. A scoping review was therefore considered the most appropriate method, as it allows for the comprehensive mapping of the existing literature, the identification of key concepts, and clarification of the range and nature of evidence available. For this reason, the review was not registered in PROSPERO, as registrations are limited to systematic reviews with predefined outcome measures. The key data extracted included patient-reported outcomes, validated QoL tools (such as P-QOL, PFDI-20, PFIQ-7, PISQ-12, and FSFI), surgical techniques used, and the duration of follow-up. The analysis was organized to compare outcomes across different study designs—including prospective cohorts, randomized controlled trials, and retrospective analyses—as well as across outcome domains, including urinary, bowel, and sexual function, and overall patient satisfaction. Recurring patterns, similarities, and important differences were identified to map the current evidence landscape and highlight existing knowledge gaps. All findings were reported descriptively, and no statistical pooling was performed due to significant clinical and methodological heterogeneity.

## 3. Results

This scoping review included 13 studies published between 2017 and 2023 that investigated patient-reported outcomes and QoL following LP. Sample sizes ranged from 15 to 102 participants, with follow-up durations varying from 3 to 28.9 months. A wide range of validated patient-reported outcome measures (PROMs) were utilized across the studies, reflecting the multifaceted impact of POP and its surgical management on patients’ lives. Pelvic floor-specific QoL measures were frequently reported, with the Pelvic Floor Distress Inventory (PFDI-20) and the Pelvic Floor Impact Questionnaire (PFIQ-7) appearing in six studies. In all cases, both PFDI-20 and PFIQ-7 scores showed statistically significant postoperative improvement.

Vo et al. reported a reduction in both PFDI-20 (from 130 to 8) and PFIQ-7 (from 148 to 10) scores within three months, and complete resolution by six months postoperatively [11]. Similarly, Yu et al. also demonstrated statistically significant improvement in these two scores after a follow-up of 15 months [17].

General prolapse-related quality of life, often assessed through the Prolapse Quality of Life (P-QOL) questionnaire, also showed significant improvement across studies. This was observed not only in single-arm studies but also in comparative designs. For instance, Khoiwal et al. reported significant postoperative gains in P-QOL, PISQ-12, and PGI-I scores in both LP and LS groups, with no statistically significant difference between them, suggesting comparable efficacy in these domains [20]. Furthermore, Astepe et al. investigated the impact of LP and sacrospinous ligament fixation (SSLF) on the QoL and sexual function of the participants postoperatively and demonstrated an improvement in P-QOL in both groups, without any significant difference between them [15]. Szymczak et al. conducted a prospective observational study showing that 75.5% of patients who underwent LP reported PGI-I improvement, along with significant improvements in PFDI-20 and PFIQ-7 (*p* < 0.04), and a reduction in EPIQ #35 (*p* < 0.001). Importantly, no urinary incontinence deterioration (assessed via the Incontinence Severity Index-ISI) or differences compared to patients in the SSLF/hysteropexy (SSHP) group were observed. A total of 68% and 65.6% of LP and SSLF patients, respectively, recommended the operative method [18].

Other domains related to urinary symptoms, such as incontinence and urinary distress, were captured using measures like the aforementioned ISI, International Consultation on Incontinence Questionnaire—Short Form (ICIQ-SF), Urogenital Distress Inventory (UDI-6), and Incontinence Impact Questionnaire (IIQ-7). In a cohort of 31 patients who underwent LP, Karslı et al. reported a significant improvement in both IIQ-7 (*p* < 0.05) and UDI-6 (*p* < 0.05) scores. Postoperative P-QOL scores also significantly improved [16]. Chao et al. conducted a retrospective cohort study with a 12-month follow-up, reporting significant improvements in UDI-6, ICIQ-SF, and POPDI-6 scores after surgery, indicating notable relief from urinary distress, incontinence symptoms, and prolapse-related discomfort [8]. Furthermore, Yang et al. conducted a prospective study evaluating the clinical benefits of LP in comparison to the gold standard technique, LS. After 12 months of follow-up, significant improvements were detected in both PFDI-20 and I-QOL scores. However, the LP group demonstrated a greater percentage decrease in both scores at 3-, 6-, and 12-month follow-ups compared to the LS group [14].

Several studies have evaluated the impact of LP on sexual function using validated instruments. Significant postoperative improvements in FSFI and PISQ-12 scores were observed in LP groups, indicating enhanced sexual health [7,13,16]. Astepe et al. reported that LP yielded significantly higher PISQ-12 scores compared to the SSLF group (*p* = 0.029) [15]. In randomized and prospective studies comparing LP with LS, such as those by Obut et al. and Khoiwal et al., both procedures resulted in significant gains in FSFI or PISQ-12 scores (*p* < 0.001), with no clear superiority between them [12,20]. Peng et al. also reported a significant postoperative improvement in PISQ-12 scores, with the LP group in which the uterus was preserved showing a statistically greater enhancement in sexual function compared to other groups [19]. Likewise, Dubinskaya et al. evaluated patients with stage 2–3 cystocele, with and without concomitant apical prolapse—treated with LP when present—and found significant postoperative improvements in sexual function, incontinence scores, and overall QoL in both subgroups [9]. Furthermore, Chao et al. found no significant difference in PISQ-IR scores between LP and LS (*p* > 0.1), suggesting comparable outcomes in sexual function [8].

## 4. Discussion

POP and POP-related symptoms such as urinary incontinence (UI), constipation or even sexual dysfunction can strongly impair patients’ QoL. Especially, women with POP and defects of the anterior compartment complain very often about disorders in their sexual life, such as reduced sexual desire and dyspareunia, leading to an impairment of the relationship between partners and a generally decreased QoL [21,22]. Nevertheless, urinary problems seem also to have a vital impact on patients’ QoL, causing many limitations on their everyday functioning [23].

Although the improvement in patients’ QoL and the regression of the preoperative symptoms are proven to be the most crucial goals of each treatment, most studies focus postoperatively only on the anatomical success [24]. For the pre- and postoperative evaluation of these aspects, numerous specific questionnaires have been developed. Regarding the assessment of women’s sexual life, the Female Sexual Function Index (FSFI) seems to be the most widely used [25], while the Pelvic Organ Prolapse/Urinary Incontinence Sexual Questionnaire (PISQ-12) is the most accurate one [26]. A revised version of PISQ-12 was lately introduced by the International Urogynecological Association (IUGA) in order to achieve a better and more detailed evaluation of the impact of POP surgery on patients’ QoL and sexuality [27].

Apical prolapse is very prevalent in patients with POP and its support plays a crucial role in achieving a durable surgical result [28]. Several methods have been introduced for treating apical prolapse, with LS being the gold standard technique [2]. However, LP seems to also be an effective and feasible alternative [29]. Across the reviewed studies, patient-reported outcomes after LP consistently demonstrated meaningful improvements, but the strength and nuance of these findings vary depending on outcome domains, study design, and follow-up duration.

Urinary function: Measures such as UDI-6, IIQ-7, ICIQ-SF, and ISI indicated a significant postoperative improvement in nearly all cohorts. Notably, Karslı et al. and Chao et al. reported reductions in urinary distress and incontinence symptoms within the first 12 months [8,16], while Szymczak et al. showed the stability of urinary outcomes beyond 24 months, confirming the durability of its benefits. Importantly, no study reported a deterioration in urinary function [18].

Sexual function: Instruments including FSFI, PISQ-12, and PISQ-IR revealed consistent postoperative gains. RCTs (e.g., Obut et al., Khoiwal et al.) demonstrated comparable improvements between LP and sacrocolpopexy [10,12], whereas observational data suggested that LP may yield superior sexual outcomes compared to sacrospinous fixation. Peng et al. highlighted that uterus-preserving LP was associated with greater postoperative sexual function scores compared to non-preserving approaches, suggesting uterine conservation may enhance patient-reported sexual health [19].

Global quality of life: QoL indices (P-QOL, PFDI-20, PFIQ-7, I-QOL) improved significantly in all studies. Comparative evidence from Yang et al. demonstrated greater gains with LP than with sacrocolpopexy, particularly in PFDI-20 and I-QOL, while smaller observational cohorts generally showed equivalence between LP and alternative techniques [14]. These findings collectively support LP as a safe and effective intervention, with outcomes at least comparable to the established gold standard.

Study design and follow-up: RCTs generally confirmed the benefits of LP but often lacked large sample sizes, whereas larger observational cohorts provided additional supportive evidence. Differences in follow-up highlight the temporal trajectory of outcomes: short-term studies (<12 months) consistently documented early symptomatic relief, while intermediate-term studies (>24 months) demonstrated sustained improvements in urinary and QoL domains without evidence of deterioration. However, long-term durability beyond three years remains insufficiently addressed.

Overall, the current evidence indicates that LP provides consistent improvements in urinary function, sexual health, and overall QoL across both randomized and observational studies. Findings from uterus-preserving procedures suggest potential additional benefits for sexual outcomes. These advantages appear to persist into the intermediate term; however, robust RCTs with larger cohorts and extended follow-up are needed to confirm durability and refine patient selection.

Several limitations must be acknowledged. Most included studies were retrospective or prospective observational designs, with only a small number of RCTs available. Differences in design, follow-up periods, and outcome measures limit direct comparability. Small sample sizes in many studies reduce generalizability, while heterogeneity in validated PROM selection complicates interpretation. The available evidence is also geographically concentrated, with the majority of studies conducted in Europe and Asia, which may limit applicability to other populations. In addition, restricting inclusion to English-language publications raises the risk of language bias, and the possibility of publication bias cannot be excluded, as studies with negative or neutral findings are less likely to be reported. Finally, the scarcity of long-term follow-up data, particularly beyond two years, prevents firm conclusions about the durability of QoL improvements.

## 5. Conclusions

According to our scoping review, LP seems to be a safe and effective method for the treatment of apical prolapse, providing not only favorable anatomical and functional outcomes but also contributing significantly to improvements in patients’ QoL and sexual health. These findings support LP as a good and feasible alternative to the gold standard LS. However, RCTs with long-term follow-up and standardized PROMs are necessary to strengthen the current evidence base and evaluate the durability of outcomes.

## Figures and Tables

**Table 1:** Data extracted of included studies about Patient-Reported Outcomes after LP.

Study (Author, Year)	Design	Sample Size (n)	Mean Follow-Up	PROMs Used	Main Findings	Overall Quality Assessment
**Erdem et al., 2022 [8]**	Cross-sectional	35	28.9 ± 5.9 months	FSFI, P-QOL	Significant improvement in all FSFI and P-QOL domains (*p* < 0.001) (Within-group comparison)	High quality
**Peng et al., 2023 [9]**	Retrospective cohort	78	12 months	PFDI-20, PFIQ-7, PISQ-12	Significantly improved postoperative (*p* < 0.05) The PISQ-12 scores in laparoscopic uterine pectopexy group were significantly higher than that in the other two groups (*p* < 0.05) (Between-group comparison)	High quality
**Astepe et al., 2019 [10]**	Comparative cohort	36 (LP)	13.1 months	PISQ-12, P-QOL	Significantly improved postoperative LP had better PISQ-12 scores compared to SSF (38.21 ± 5.69 vs. 36.86 ± 3.15) but similar P-QOL scores (Between-group comparison)	High quality
**Obut et al., 2021 [11]**	Prospective randomized	32 (LP)	12 months	FSFI, P-QOL	Significant improvement in all FSFI and P-QOL domains in both LP and laparoscopic sacrohysteropexy groups (*p* < 0.001) (Within-group and Between-group comparison)	High quality
**Khoiwal et al., 2022 [12]**	RCT	15 (LP)	6 months	PGI-I, P-QOL, PISQ-12	Significant improvement in all domains in LP and LS groups PGI-I (*p* = 0.006 vs. 0.009) PISQ-12 (*p* < 0.001 in both groups) P-QOL(*p* < 0.001 in both groups)—no difference between the two groups (Within-group and Between-group comparison)	High quality
**Karslı et al., 2021 [13]**	Prospective	31	3 months	P-QOL, PISQ-12, UDI-6, IIQ-7	Significant improvement in all domains (*p* =0.001) (Within-group comparison)	High quality
**Tahaoglu et al., 2017 [14]**	Observational	22	10.4 months	FSFI, P-QOL	Significant improvement in all FSFI and P-QOL domains (*p* < 0.05) (Within-group comparison)	High quality
**Yu et al., 2023 [15]**	Prospective	49	15 months	PFDI-20, PFIQ-7	Significant improvement in all FSFI and P-QOL domains (*p* < 0.001) (Within-group comparison)	High quality
**Szymczak et al., 2022 [16]**	Prospective observational	53	26.9 ± 12 months	PGI-I, ISI, EPIQ #35, PFIQ-7, PFDI-20	PGI-I: postoperative improvement in 75.5% of the LP patients and 44 (72.1%) SSLF patients. Improvement in PFDI-20 and PFIQ-7 (*p* < 0.04) EPIQ #35 was significantly reduced (*p* < 0.001) ISI: no postoperative deterioration of UI symptoms (Within-group comparison) All scores did not differ between the LP and SSLF/SSHP groups. (Between-group comparison)	High quality
**Chao et al., 2022 [17]**	Retrospective cohort	30 (LP)	12 months	UDI-6, ICIQ-SF, POPDI-6, PISQ-IR	UDI-6, ICIQ-SF, and POPDI-6 improved significantly (Within-group comparison) No significant differences in the mean difference in postoperative and preoperative UDI-6 (*p* = 0.834), ICIQ-SF (*p* = 0.861), POPDI-6 (*p* = 0.775) scores between LP and LS groups. No significant differences in the mean difference in postoperative and preoperative PISQ-IR question 9 (*p* = 0.351), PISQ-IR question 11 (*p* = 0.715), PISQ-IR question 18 (*p* = 0.192), or PISQ-IR question 19a (*p* = 0.106) between the two groups. (Between-group comparison)	High quality
**Yang et al., 2023 [18]**	Prospective cohort	102 (LP)	12 months	PFDI-20, I-QOL	PFDI-20, I-QOL scores had improved significantly LP group showed a greater percent decrease in PFDI-20 and greater percent increase in I-QOL from baseline compared to LS group (Between-group comparison)	High quality
**Vo et al., 2023 [19]**	Retrospective case series	58	6 months	PFDI-20, PFIQ-7	PFDI-20 from 130 → 8 and PFIQ-7 score 148 → 10 at 3 months; 0 at 6 months for both scores (Within-group comparison)	Moderate quality
**Dubinskaya et al., 2022 [20]**	Prospective	22	12 months	ICIQ-UI-SF, ICIQ-V, PGI-I	All scores improved significantly (*p* < 0.001) (Within-group comparison)	High quality

FSFI = Female Sexual Function Index; P-QOL = Prolapse Quality of Life; PFDI-20 = Pelvic Floor Distress Inventory; PFIQ-7 = Pelvic Floor Impact Questionnaire; PISQ-12 = Pelvic Organ Prolapse/Urinary Incontinence Sexual Questionnaire; PGI-I = Patient Global Impression of Improvement; ISI = Incontinence Severity Index; ICIQ-SF = International Consultation on Incontinence Questionnaire—Short Form; UDI-6 = Urogenital Distress Inventory; IIQ-7 = Incontinence Impact Questionnaire; EPIQ = Epidemiology of Prolapse and Incontinence Questionnaire; I-QOL = Incontinence Quality of Life; LS: laparoscopic sacrocolpopexy; LP: laparoscopic pectopexy, PISQ-IR: Pelvic Organ Prolapse/Urinary Incontinence Sexual Questionnaire, IUGA-Revised, POPDI-6: Pelvic organ Prolapse Distress Inverntory-6.

## Data Availability

Not applicable.

## Supplementary Materials

The following supporting information can be downloaded at: https://www.mdpi.com/article/10.3390/jcm14176318/s1, Table S1: PRISMA-ScR; Table S2: JBI Critical Appraisal Checklist.

## Abbreviations

The following abbreviations are used in this manuscript:

POP	Pelvic organ prolapse
LS	laparoscopic sacrocolpopexy
LP	Laparoscopic pectopexy
PROs	patient-reported outcomes
QoL	quality of life
FSFI	Female Sexual Function Index
P-QOL	Prolapse Quality of Life
PFDI-20	Pelvic Floor Distress Inventory
PFIQ-7	Pelvic Floor Impact Questionnaire
PISQ-12	Pelvic Organ Prolapse/Urinary Incontinence Sexual Questionnaire
PGI-I	Patient Global Impression of Improvement
ISI	Incontinence Severity Index
ICIQ-SF	International Consultation on Incontinence Questionnaire—Short Form
UDI-6	Urogenital Distress Inventory
IIQ-7	Incontinence Impact Questionnaire
EPIQ	Epidemiology of Prolapse and Incontinence Questionnaire
I-QOL	Incontinence Quality of Life
PISQ-IR	Pelvic Organ Prolapse/Urinary Incontinence Sexual Questionnaire IUGA-Revised
POPDI-6	Pelvic organ Prolapse Distress Inverntory-6
UI	Urinary incontinence
IUGA	International Urogynecological Association
RCTs	Randomized controlled trials
SSHP	Sacrospinous ligament hysteropexy
SSLF	Sacrospinous ligament fixation
