# Supplementary material for: Patient-Reported Outcomes and Quality of Life After Laparoscopic Pectopexy
Source: J Clin Med. 2025 Sep 7;14(17):6318. doi: 10.3390/jcm14176318 (PMC12428886; doi:10.3390/jcm14176318)
Supplement: Supplementary file 1 [file jcm-14-06318-s001.zip › Table S2--JBI Pectopexy 26.06 - Foglio1.pdf]

Title	Author, Year	D1	D2	D3	D4	D5	D6	D7	D8
Effectiveness of Laparoscopic Pectopexy for Pelvic Organ Prolapse Compared with Laparoscopic Sacrocolpopexy	Yingying Yang et al, 2023	YES	YES	YES	YES	YES	YES	YES	YES
Comparison of the Quality of Life and Female Sexual Function Following Laparoscopic Pectopexy and Laparoscopic Sacrohysteropexy in Apical Prolapse Patients	Mehmet Obut ET AL., 2021	YES	YES	YES	YES	YES	YES	YES	YES
Intermediate-term outcomes of laparoscopic pectopexy and vaginal sacrospinous fixation: a comparative study	Bahar Sariibrahim Astepe et al., 2019	YES	YES	YES	YES	YES	YES	YES	YES
Comparison of efficacy between laparoscopic pectopexy and laparoscopic high uterosacral ligament suspension in the treatment of apical prolapse-short term results	Juan Peng et al., 2023	YES	YES	YES	YES	YES	YES	YES	YES
Intermediate-term follow-up of laparoscopic pectopexy cases and their effects on sexual function and quality of life: a cross-sectional study	Selami Erdem, 2022	UNCLEAR	YES	YES	YES	YES	YES	YES	YES
Initial evaluation treatment results of laparoscopic pectopexy in the management of uterine prolapse	Vo Phi Long et al., 2023	UNCLEAR	UNCLEAR	YES	YES	UNCLEAR	YES	YES	YES
Comparison of laparoscopic pectopexy with the standard laparoscopic sacropexy for apical prolapse: an exploratory randomized controlled trial	Kavita Khoiwal, 2022	YES	YES	YES	YES	YES	YES	YES	YES
Laparoscopic pectopexy with native tissue repair for pelvic organ prolapse	Pei Yu et al., 2023	YES	YES	YES	YES	YES	YES	YES	YES
Perioperative and Long-Term Anatomical and Subjective Outcomes of Laparoscopic Pectopexy and Sacrospinous Ligament Suspension for POP-Q Stages II–IV Apical Prolapse	Paulina Szymczak et al. 2022	YES	YES	YES	YES	YES	YES	UNCLEAR	YES
Efficacy of minimally invasive pectopexy with concomitant I-stop-mini sling for women with pelvic organ prolapse and overt stress urinary incontinence: A retrospective cohort study	Wei-Ting Chao et al. 2023	YES	YES	YES	YES	YES	YES	YES	YES
